# How Accurate Are Veterinary Clinicians Employing Flexicult Vet for Identification and Antimicrobial Susceptibility Testing of Urinary Bacteria?

**DOI:** 10.3390/antibiotics10101160

**Published:** 2021-09-24

**Authors:** Blaž Cugmas, Miha Avberšek, Teja Rosa, Leonida Godec, Eva Štruc, Majda Golob, Irena Zdovc

**Affiliations:** 1 Veterinary Clinic Zamba, Vets4science d.o.o., 3000 Celje, Slovenia; miha@vets4science.com (M.A.); teja.rosa@gmail.com (T.R.); leonida.kajdic@gmail.com (L.G.); 2 Biophotonics Laboratory, Institute of Atomic Physics and Spectroscopy, University of Latvia, 1586 Riga, Latvia; 3 Vetamplify SIA, Veterinary Services, 1009 Riga, Latvia; eva@vetamplify.com; 4 Institute of Microbiology and Parasitology, Veterinary Faculty, University of Ljubljana, 1000 Ljubljana, Slovenia; majda.golob@vf.uni-lj.si (M.G.); irena.zdovc@vf.uni-lj.si (I.Z.)

**Keywords:** urinary tract infection, Flexicult Vet, antimicrobial susceptibility testing, pathogen identification, dogs, cats, veterinary microbiology

## Abstract

Antibiotics are frequently used for treating urinary tract infections (UTI) in dogs and cats. UTI often requires time-consuming and expensive antimicrobial susceptibility testing (AST). Alternatively, clinicians can employ Flexicult Vet, an affordable chromogenic agar with added antibiotics for in-clinic AST. We investigated how well veterinary microbiologists and clinicians, without any prior experience, employ Flexicult Vet for the identification and AST of the most common canine and feline urinary pathogenic bacteria. We prepared 47 monoculture plates containing 10 bacterial species. The test’s mean accuracy was 75.1% for bacteria identification (84.6% and 68.7% for microbiologists and clinicians, respectively) and 79.2% for AST (80.7% and 78.2%). All evaluators employed Flexicult Vet with the accuracies over 90% for the distinctively colored bacteria like *Escherichia coli* (red), *Enterococcus faecalis* (turquoise), and *Proteus* spp. (pale brown). However, the evaluators’ experience proved important in recognizing lightly colored bacteria like *Staphylococcus pseudintermedius* (accuracies of 82.6% and 40.3%). Misidentifications of *E. faecium* additionally worsened AST performance since bacterial intrinsic resistance could not be considered. Finally, only 33.3% (3/9) of methicillin-resistant *S. pseudintermedius* (MRSP) were correctly detected. To conclude, Flexicult Vet proved reliable for certain urinary pathogens. In contrast, light-colored bacteria (e.g., *Staphylococcus*), often misidentified, require a standard AST.

## 1. Introduction

Urinary tract infections (UTIs) are common in small animals since up to 27% of dogs, especially females, are affected during their lifetime. In cats, UTIs are rarer (<2%) and they usually appear in older cats (>10 years) [1,2,3]. Uncomplicated UTI can sporadically happen in otherwise healthy animals. In contrast, urinary infections in pets with anatomic or functional abnormalities may often persist, reoccur, or be insensitive to treatment. In 85% of cases, a single pathogen is the main cause of UTI. The most frequently isolated species are *Escherichia coli* (>50%), followed by *Staphylococcus* spp., *Enterococcus* spp., *Streptococcus* spp., *Proteus* spp., *Enterobacter* spp., *Pseudomonas* spp., and *Klebsiella* spp. [1,2,4,5,6,7,8].

Due to its high incidence, bacterial UTI is one of the main reasons for prescribing antibiotics in small animal medicine [9]. In contrast to human medicine, the range of available veterinary antibiotics is limited; thus, special care is required by veterinary clinicians to prevent misuse or overuse of antibiotics and to avoid the appearance of resistant strains. Resistant bacteria are an important but not the only undesirable outcome of improper use of antibiotics. Animal health (due to drug side effects, normal flora distortions [8,10,11]) and treatment costs (side effects and prolonged or recurrent UTIs) can all be directly impacted. Since antimicrobial resistance can also affect the health of humans (e.g., due to animal–human transmissions [12]), other animals, and environment, correct antibiotic use for UTI can contribute considerably to the One Health approach [13].

Therefore, managing UTI often requires antimicrobial susceptibility testing (AST) [1,3]. However, AST according to the CLSI standards [14] based on disc diffusion, broth dilution, or agar dilution methods in the certified microbiological laboratories can be time-consuming (up to a week) and expensive for some pet owners. Moreover, sample storage and shipping additionally contribute to the uncertainty of the final results [7]. Thus, empirical antimicrobial treatment is still the most comfortable for clinicians in small animal practice, who frequently opt even for second-line antibiotics (in 57% of UTI cases) [15].

**Figure 1:**
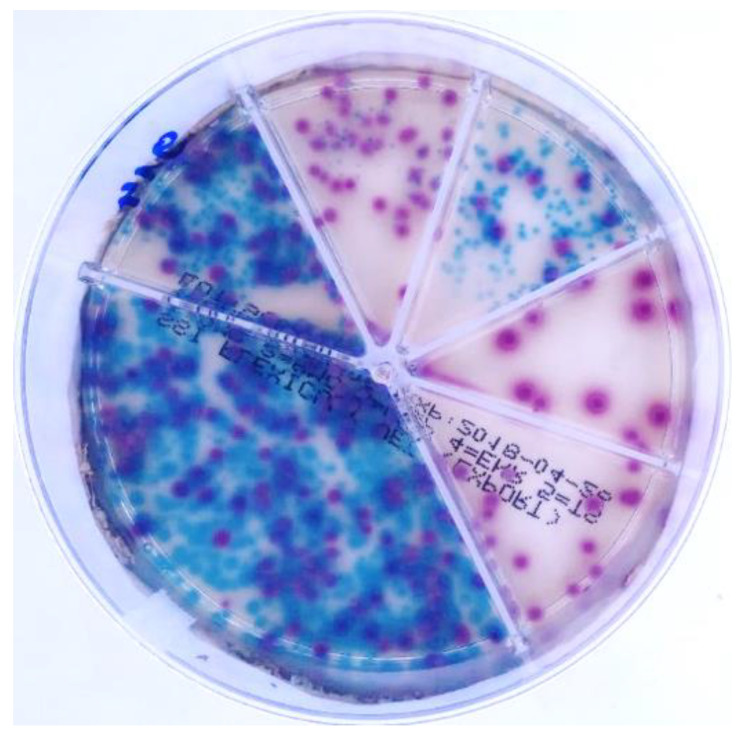
*Escherichia coli* (red colonies) and *Enterococcus faecalis* (turquoise) on Flexicult Vet agar.

Point-of-care (POC) tests have recently appeared to provide a faster and cheaper in-clinic AST, which might reduce the utilization of unnecessary or inappropriate antibiotics [16]. One of the most popular is Flexicult Vet, based on a chromogenic nonselective culture medium with added antibiotics in separate compartments (Figure 1). The test promises to provide data about bacteria species and sensitivity to the most common antibiotics in only 24 h. Existing studies indicated that the evaluator’s experience plays an important role in the test’s performance and accuracy. For example, one expert reached an accuracy of 100% using Flexicult Vet for bacterial identification [4]. On the other hand, less experienced evaluators achieved the lower accuracies of 53% [4], 58–77% [17], and 92–98% [18]. Furthermore, the test’s AST performance resulted in accuracies between 39 and 99% [4,17,18]. 

Due to the reported large differences in Flexicult Vet performance, the aim of the present study was to evaluate how well the potential end-users, i.e., veterinary clinicians without a microbiological background, had employed Flexicult Vet for bacterial identification and AST interpretation. First, we inoculated Flexicult Vet with the monocultures of the most frequent canine and feline urinary pathogens. Furthermore, we compared how accurate bacteria were identified, and AST interpreted by experts (microbiologists and microbiological assistants) or veterinary practitioners, all without any prior Flexicult Vet experience. The results pointed out that veterinary clinicians can benefit from Flexicult Vet in some cases, but many limitations remain. 

## 2. Results

**Figure 2:**
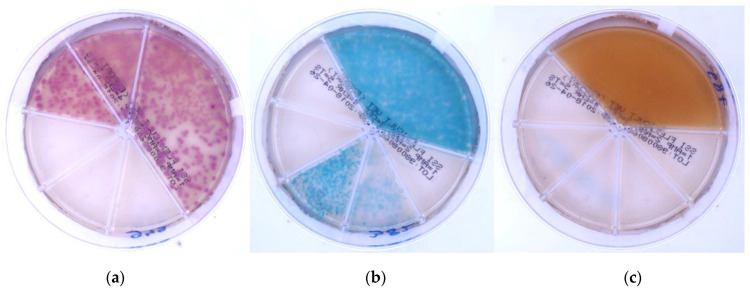
Bacteria of (**a**) *Escherichia coli* (red), (**b**) *Enetrococcus faecalis* (turquoise), and (**c**) *Proteus* spp. (brown), exhibiting distinct colors on the Flexicult Vet agar.

On average, 75.1% of samples were identified correctly (Table 1). Experts outperformed clinicians with the mean bacteria species identification accuracies of 84.6% versus 68.7%, respectively. Moreover, clinicians seemed less confident in their evaluations due to the slightly wider 95% confidence interval (CI) (18.2 versus 14.7 percentage points, respectively). Surprisingly, not a single bacterium was identified perfectly. The highest identification accuracies were expectedly achieved for bacteria with distinct colors like red (*Escherichia coli*, 90.0%), turquoise (*Enterococcus faecalis*, 97.8%), and pale brown (*Proteus* spp., 90.0%) (Figure 2). Additionally, nine raters correctly identified a single isolate of *Pseudomonas aeruginosa* (an accuracy of 90.0%).

**Figure 3:**
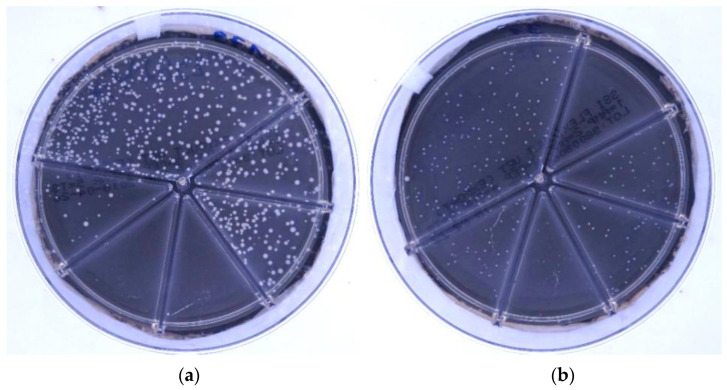
Pale-looking bacteria of (**a**) *Staphylococcus pseudintermedius* and (**b**) *Enterococcus faecium* on the Flexicult Vet agar. For display purposes, the agars were photographed with a dark background.

Oppositely, identification was more challenging for light-colored (pale) colonies (Figure 3). We found the lowest identification accuracy for *Enterococcus faecium* (29.0%), which was mostly misidentify for *Staphylococcus pseudintermedius* (35.0% of *E. faecium* samples) and *Streptococcus canis* (25.9%). Identifying *S. pseudintermedius* resulted in the highest discrepancy between experts and clinicians (82.6% vs. 40.3%), who had mistaken *S. pseudintermedius* for *E. faecium* and *S. canis* in 21.6% and 22.9% of cases, respectively.

In comparison with the bacterial identification, antimicrobial susceptibility testing (AST) achieved a slightly better mean accuracy of 79.2% (Table 2). Additionally, AST performance by experts or clinicians was comparable. Flexicult Vet enabled accurate AST results for enrofloxacin (ENR, 88.7%) and bacterial species of *E. coli* and *E. faecalis* (>90.0%). Oppositely, the test performed poorly with the accuracies below 50% for *Proteus* spp. (for all antibiotics) and *S. pseudintermedius* (for penicillin group: ampicillin—AMP; amoxicillin —AMC; oxacillin—OXA). Alarmingly, only 33.3% of methicillin-resistant *S. pseudintermedius* (MRSP) were detected. A very low accuracy (30.0%) was also achieved for *E. faecium* sensitivity to trimethoprim/sulfamethoxazole (STX). A majority (>70%) of AST misestimates happened due to the *Enterococcus* spp. intrinsic resistance to STX, which was either forgotten or discarded since bacteria species were misidentified. 

## 3. Discussion

Point-of-care (POC) microbiological tests like Flexicult Vet could improve antibiotics use since they offer identification and antimicrobial susceptibility testing (AST) of UTI-causing bacteria. To the best of our knowledge, there are no studies that compared the performance of experts and clinicians in using microbiological POC tests on the controlled monoculture samples. The recent field studies with real urine samples [4,17,18], which included experts and beginners, showed that Flexicult Vet enabled identification of bacteria with an accuracy between 53 and 100%, which is in line with the accuracy of 75.1%, reported in this study. However, evaluator experience plays an important role in the test’s performance. Although all evaluators handled Flexicult Vet for the first time, microbiological experts outperformed clinicians in bacteria identification for 15.9 percentage points (accuracies of 84.6% vs. 68.7%). The difference between evaluators was significantly smaller than the one reported by Guardabassi et al. [4], where a beginner recognized only 53% of samples, contrary to the flawless expert (100%). Experts from the other studies [17,18] also achieved an excellent identification accuracy (>97%), which was significantly higher than the one reached by microbiological evaluators in our study (84.6%). However, all other studies included only a single expert evaluator, well familiar with Flexicult Vet, in contrast to the experts in this study, who met Flexicult Vet for the first time.

In general, all evaluators in this study identified colorful bacteria very well (accuracies of >90.0%) (Figure 2). Oppositely, identification of light-colored bacteria was unreliable (Figure 3, *S. pseudintermedius*, accuracy of 57.2%, *E. faecium*, 29.0%). The pale colonies were often recognized as *S. canis*. The mentioned misidentifications could be partially addressed by a prolonged incubation time of 48 h, enabling colonies to develop more characteristic color. Additionally, evaluators should pay more attention to colony size. On Flexicult Vet, *S. pseudintermedius* exhibits moderately sized colonies, but *S. canis* develops only microcolonies.

Recognizing bacteria well is especially important for assuring a high AST accuracy. For example, *E. faecium*, which has an intrinsic resistance to STX, was misidentified in 71% of cases. Since 5 (out of 6) samples did not exhibit any growth in the STX compartment, the clinician could falsely choose STX as an antibiotic of choice. Furthermore, in one *E. faecium* sample, three clinicians and one expert forgot to consider its intrinsic resistance to STX, despite correctly recognizing the strain. As intrinsic resistance also concerns penicillin antibiotics (e.g., *Proteus vulgaris, Pseudomonas aeruginosa*), Flexicult Vet could be supplemented with a special AST-deploying protocol, reminding users of a possibility of intrinsic resistance.

**Figure 4:**
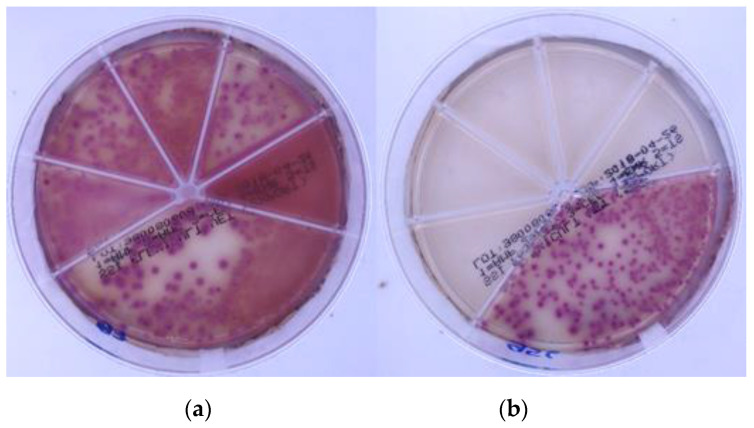
*Escherichia coli* on Flexicult Vet. The strains were falsely interpreted as (**a**) sensitive (*S*) or (**b**) resistant (*R*) to antibiotics.

Neglecting intrinsic resistance was not the only user error detected. In certain cases (Figure 4), clinical evaluators interpreted growing bacteria as sensitive. Oppositely, the absence of growth led to labeling bacterium as resistant. We speculate that these errors could happen due to mixing up *R* (resistant) and *S* (sensitive) labels when filling the AST results form. We assume that similar administrative mistakes could be even more common when evaluators were in the (often noisy and hectic) clinical environment.

In general, Flexicult Vet provided a decent AST for *E. coli* (accuracy of >91.5%) and *E. faecalis* (>86.2%). Despite good identification, poor AST results were achieved for *Proteus* spp. In general, we detected many false sensitive strains (Table 2), which could indicate high antibiotic concentrations. Obviously, appropriate antibiotic concentrations cannot be guaranteed in a single POC test for all bacteria since UTI pathogens (especially *Staphylococcus* spp. versus others) have different AST breakpoints.

The purpose of oxacillin in Flexicult Vet is the detection of methicillin-resistant *S. pseudintermedius* (MRSP). In over a decade, the number of canine MRSP strains in Slovenia has been steadily rising. Moreover, the multidrug-resistant isolates to five or more antimicrobial groups, including oxacillin, penicillin, clindamycin, erythromycin, and trimethoprim, are prevalent [19]. If AST results allow, clinicians often rely on doxycycline for MRSP infection treatment.

Our study included 9 MRSP strains (in addition to one methicillin-sensitive strain). Initially, clinical evaluators had problems recognizing the species since they misidentified 43.8% samples. Additionally, two thirds (6/9) of MRSPs were falsely perceived as sensitive, which led to a conclusion that the OXA concentration is too high. However, this is not in agreement with Guardabassi et al. who showed that 0.125 µg/mL of OXA was the most suitable for cultivating MRSPs and suppressing a methicillin-susceptible *S. pseudintermedius*. The study demonstrated [4] that the larger OXA concentrations, including the CLSI breakpoint (i.e., R ≥ 0.5 µg/mL [14]), suppressed between 27 and 40% of MRSPs.

## 4. Materials and Methods

We tested a commercially available POC Flexicult Vet Scandinavia (SSI Diagnostica, Hillerød, Denmark) for the identification and AST of UTI-causing bacteria in dogs and cats. Briefly, Flexicult Vet includes the modified chromogenic Müller-Hinton II agar (MH II). The Petri dish was divided into six compartments; one big without antibiotics and five smaller compartments with undisclosed concentrations of ampicillin (AMP), amoxicillin/clavulanate (AMC), oxacillin (OXA), enrofloxacin (ENR), and trimethoprim/sulfamethoxazole (SXT). Bacterial identification is based on the color, shape, and diameter of colonies (CFUs), while the absence or presence of bacterial growth can determine susceptibility to antibiotics (AST). The number of CFUs in the big compartment additionally allows semi-quantitative determination of bacterial concentration in urine, which can reveal clinically relevant bacteriuria due to its correlation with the urine sampling techniques (i.e., free catch, cystocentesis, and catheter specimen thresholds are ≥10^5^, ≥10^3^, and ≥10^4^ CFU/mL, respectively) [4].

The monoculture suspension samples were prepared in a laboratory using 47 common canine and feline UTI strains from the internal bacterial collection at the Institute of Microbiology and Parasitology, Veterinary Faculty, University of Ljubljana. The samples included *E. coli* (13 strains), *S. pseudintermedius* (11, including 9 phenotypically and genetically identified as methicillin-resistant *S. pseudintermedius*, MRSP), *E. faecalis* (9), *E. faecium* (6), *Proteus vulgaris* (2), *Proteus mirabilis* (2), *Klebsiella pneumoniae* (1), *Enterobacter cloacae* (1), *Enterobacter aerogenes* (1), and *P. aeruginosa* (1). At least one reference strain with a known antimicrobial activity was used for each bacterial group, *E. coli* ATCC 25922, *S. aureus* ATCC 29213, *E. faecalis* ATCC 29212, *P. aeruginosa* ATCC 27853, *Klebsiella pneumoniae* ATCC 51503, and *Proteus mirabilis* DSM 788. Other strains were obtained from the different proficiency test trials and clinical isolates. For all strains, we performed AST based on a microdilution method (Sensititre, Thermo Fisher Scientific Inc, Waltham, Massachusetts, USA) or disk diffusion method according to the CLSI standard [14,20]. Bacteria represented by a single sample were joined into a group of *Others*. For a straightforward comparison with Flexicult Vet, intermediate samples were considered as resistant (*R*).

**Figure 5:**
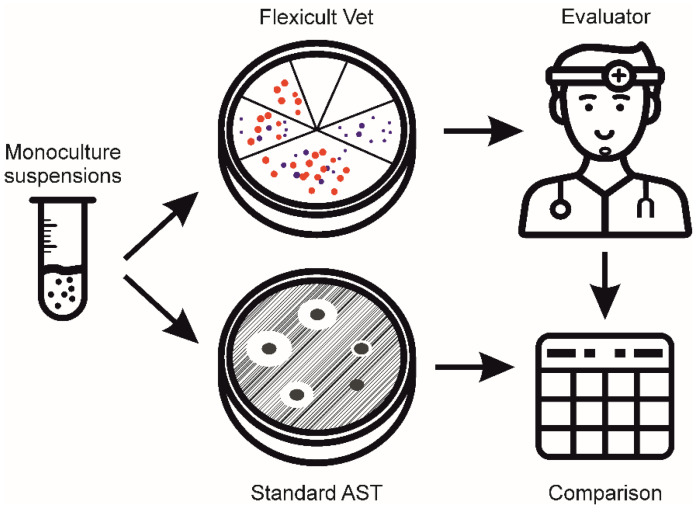
Veterinary microbiological experts and veterinary clinicians (Evaluators) performed an identification and antimicrobial susceptibility testing (AST) of UTI bacteria growing on Flexicult Vet plates. The results were compared to the standard AST.

Monocultures of bacterial suspensions were prepared with various concentrations (10^4^, 10^5^, and 10^6^ CFU/mL) in sterile saline and inoculated onto Flexicult Vet plates according to the manufacturer’s instructions. After the incubation (24 h at 35 °C), 10 participants without any prior Flexicult Vet experience evaluated the plates (Figure 5). There were four *expert* evaluators, microbiologists and microbiology lab assistants in a veterinary microbiological laboratory. Additionally, six veterinary *clinicians* were involved.

Before the evaluations, we briefly introduced Flexicult Vet to the evaluators. We started with an oral presentation. On a few examples, we additionally demonstrated how to identify bacteria and interpret the plate to obtain AST. First, an evaluator had to provide a bacteria species. In case of doubt, they could list up to three species if selected species were supposedly not crucial for an AST performance. Secondly, the susceptibility (*S*) or resistance (*R*) for each antibiotic was retrieved. The final strain score was calculated as a mean of all evaluators’ scores. We calculated confidence intervals (*CI*) as

(1) $$CI=\bar{x}\pm \frac{SD\cdot q}{\sqrt{n}}$$

where $\bar{x}$ and *SD* are the mean and standard deviation of evaluator scores, *n* is a number of evaluator scores, and *q* is a quantile (i.e., the left-tailed inverse of the Student’s t-distribution with the probability of 0.975 and the degree of freedom of *n* − 1). All calculations were done in the Excel program (Microsoft Excel 2016, 16.0, Microsoft, Redmond, WA, USA). In the end, species and antibiotic score means and confidence intervals were arranged in a tabular form. Plates were photographed by a lightbox (Petriview Box, Vets4science d.o.o., Celje, Slovenia, www.petriview.net, accessed on 1 August 2021).

## 5. Conclusions

Flexicult Vet could be a promising POC test for detecting, identifying, and AST of UTI-causing bacteria. However, to obtain the optimal test performance, which can decrease inappropriate antibiotic use and bacterial resistance, evaluators need to be properly trained; in performing and interpreting Flexicult Vet. Evaluators in this study, regardless of experience, employed the test well for colorful bacteria like *E. coli* and *E. faecalis*. However, experience played an important role in recognizing light-colored bacteria, which can crucially affect the AST accuracy. The study also showed that users could be negligent in considering bacterial intrinsic resistance or selecting R/S labels. Finally, many undiscovered MRSP strains require further studies with *S. pseudintermedius*. Despite the drawbacks mentioned, Flexicult Vet could be useful for veterinary clinicians when dealing with UTI, especially when a pet owner is not willing to cover laboratory AST expenses.

## Figures and Tables

**Table 1:** Mean and 95% confidence intervals (CI, squared brackets) of identification accuracy (%) retrieved by experts (E) and clinicians (C). The most frequent misidentified bacteria are listed in the rounded brackets. Bacteria are abbreviated as *Enterobacter* spp. (Es), *Klebsiella* spp. (Ks), *S. canis* (Sc), *S. aureus* (Sa), and *P. aeruginosa* (Pa).

		Flexicult Vet					
True Species	*Investigator*	*E. coli*	*S. pseudint.*	*E. faecium*	*E. faecalis*	*Proteus* spp.	Other
*E. coli, n* = 13	E	**98.1** [95.2–100.0]	1.9				
	C	**84.6** [72.7–96.6]	12.8	1.3			1.3 (Es, Ks)
	All	**90.0** [82.3–98.7]	8.5	0.8			0.8 (Ea, Ks)
*S. pseudintermedius, n* = 11	E	8.3	**82.6** [68.3–96.9]	3.4			5.7 (Sc, Sa)
	C	7.6	**40.3** [22.8–57.7]	21.6			30.6 (Sc, Pa)
	All	7.9	**57.2** [44.1–70.3]	14.3			20.6 (Sc, Sa, Pa)
*E. faecium, n* = 6	E		50.0	**31.3** [15.2–47.3]			18.8 (Sc)
	C	1.6	25.0	**27.6** [18.8–36.3]	5.1		40.8 (Sc, Pa, Ks)
	All	1.0	35.0	**29.0** [21.7–36.3]	3.1		32.0 (Sc, Pa, Ks)
*E. faecalis, n* = 9	E			1.4	**98.6** [95.4–100]		
	C			0.9	**97.2** [94.0–100]		1.9 (Pa)
	All			1.1	**97.8** [95.0–100]		1.1 (Pa)
*Proteus* spp., *n* = 4	E					**93.8** [73.9–100]	6.3 (Pa)
	C				8.3	**87.5** [62.1–100]	4.2 (Pa)
	All				5.0	**90.0** [71.6–100]	5.0 (Pa)
Other, *n* = 4	E	8.3			3.1		**88.5** [63.5–100]
	C	2.1	4.9	3.5	9.7		**79.9** [52.0–100]
	All	4.6	2.9	2.1	7.1		**83.3** [57.6–100]
All, *n* = 47		Experts: **84.6** [77.2–91.9]		Clinicians: **68.7** [59.6–77.8]		All: **75.1** [67.4–82.8]	

**Table 2:** Absolute sample counts and AST accuracy (in %, means and 95% confidence intervals, CI, in the squared brackets) for Flexicult Vet, evaluated by experts (E) and clinicians (C). Antibiotic abbreviations are the following: ampicillin (AMP), amoxicillin (AMC), oxacillin (OXA), enrofloxacin (ENR), trimethoprim/sulfamethoxazole (SXT). * denotes a group with one sample less.

Bacteria	Evaluator	AST	Flexicult Vet									
			AMP		AMC		OXA		ENR		SXT	
			R	S	R	S	R	S	R	S	R	S
*E. coli, n* = 13	E	R	9	1	9	1			6	1	4	1
		S	–	3	–	3			–	6	–	8
	C	R	8.83	1.17	9	1			6	1	4	1
		S	0.17	2.83	–	3			–	6	0.17	7.83
	All		90.8 [74.1–100]		92.3 [75.5–100]				92.3 [75.5–100]		91.5 [74.8–100]	
*S. pseudintermedius, n* = 10	E	R	6.75	2.25	2.50	6.50	3.13	5.88	8.75	0.25	9	–
		S	–	–	–	1	–	1	–	1	–	1
	C	R	7	2	2.17	6.83	3	6	8.83	0.17	8.83	0.17
		S	–	–	–	1	–	1	0.17	0.83	–	1
	All		76.7 * [53.0–100]		33.0 [2.3–63.7]		40.5 [11.7–69.3]		97.0 [92.2–100]		99.0 [96.7–100]	
*E. faecium, n* = 6	E	R	2	–	2	–			2	–	1.50	4.50
		S	–	4	–	4			–	4	–	–
	C	R	1.63	0.37	1.63	0.37			1.80	0.20	2	4
		S	1.27	2.73	–	4			0.93	3.07	–	–
	All		83.7 [77.7–89.7]		96.3 [90.3–100]				88.7 [80.6–96.7]		30.0 [7.9–52.1]	
*E. faecalis, n* = 9	E	R	–	–	–	–			5	–	8.50	0.50
		S	–	8	–	8			1	2	–	–
	C	R	–	–	–	–			4.83	0.17	8.17	0.83
		S	0.17	7.83	–	8			1	2	–	–
	All		98.7 * [95.8–100]		100 * [100–100]				86.2 * [57.0–100]		92.2 [84.4–99.7]	
*Proteus* spp., *n* = 4	E	R	2.25	1.75	–	2			–	2	–	2
		S	–	–	0.75	1.25			–	2	0.75	1.25
	C	R	1.17	2.83	–	2			–	2	–	2
		S	–	–	–	2			–	2	0.33	1.67
	All		40.0 [0–100]		42.5 [0–100]				50.0 [0–100]		37.5 [0–100]	
Other, *n* = 4	E	R	2.13	0.88	1	1			1	–	–	–
		S	–	–	–	1			–	3	0.13	2.88
	C	R	2.08	0.92	1.22	0.78			1	–	–	–
		S	–	–	0.33	0.67			–	3	0.42	2.58
	All		70.0 * [5.4–1]		64.4 * [0–100]				100 [100–100]		90.0 * [77.6–100]	
All samples *n* = 43 (AMP), 44 (AMC), 10 (OXA), 45 (ENR), 45 (SXT)	All E C		82.1 [73.3–90.9] 86.3 [76.6–96.1] 79.3 [70.5–88.1]		74.4 [62.1–86.6] 74.4 [62.0–86.9] 74.3 [61.9–86.7]		40.6 [11.8–69.4] 41.3 [9.7–72.9] 40.2 [12.0–68.4]		88.7 [80.1–97.3] 90.6 [81.9–99.2] 87.4 [78.8–96.1]		80.2 [70.4–90.0] 80.3 [70.0–90.6] 80.2 [70.4–89.9]	
All together (*n* = 187)					All: 79.2 [74.2–84.2]		E: 80.7 [75.5–85.9]		C: 78.2 [73.2–83.2]			

## Data Availability

Data available on request.
